# Seamless editing of the chloroplast genome in plants

**DOI:** 10.1186/s12870-016-0857-6

**Published:** 2016-07-29

**Authors:** Elena Martin Avila, Martin F. Gisby, Anil Day

**Affiliations:** 1 Faculty of Life Sciences, The University of Manchester, Manchester, M13 9PT UK; 2 Present address: Research School of Biology, The Australian National University, Acton, ACT 2601 Australia

**Keywords:** Overlapping genes, Rubisco LS mutation, Organelle genome editing, Homologous recombination, Translational coupling

## Abstract

**Background:**

Gene editing technologies enable the precise insertion of favourable mutations and performance enhancing trait genes into chromosomes whilst excluding all excess DNA from modified genomes. The technology gives rise to a new class of biotech crops which is likely to have widespread applications in agriculture. Despite progress in the nucleus, the seamless insertions of point mutations and non-selectable foreign genes into the organelle genomes of crops have not been described. The chloroplast genome is an attractive target to improve photosynthesis and crop performance. Current chloroplast genome engineering technologies for introducing point mutations into native chloroplast genes leave DNA scars, such as the target sites for recombination enzymes. Seamless editing methods to modify chloroplast genes need to address reversal of site-directed point mutations by template mediated repair with the vast excess of wild type chloroplast genomes that are present early in the transformation process.

**Results:**

Using tobacco, we developed an efficient two-step method to edit a chloroplast gene by replacing the wild type sequence with a transient intermediate. This was resolved to the final edited gene by recombination between imperfect direct repeats. Six out of 11 transplastomic plants isolated contained the desired intermediate and at the second step this was resolved to the edited chloroplast gene in five of six plants tested. Maintenance of a single base deletion mutation in an imperfect direct repeat of the native chloroplast *rbcL* gene showed the limited influence of biased repair back to the wild type sequence. The deletion caused a frameshift, which replaced the five C-terminal amino acids of the Rubisco large subunit with 16 alternative residues resulting in a ~30-fold reduction in its accumulation. We monitored the process in vivo by engineering an overlapping *gusA* gene downstream of the edited *rbcL* gene. Translational coupling between the overlapping *rbcL* and *gusA* genes resulted in relatively high GUS accumulation (~0.5 % of leaf protein).

**Conclusions:**

Editing chloroplast genomes using transient imperfect direct repeats provides an efficient method for introducing point mutations into chloroplast genes. Moreover, we describe the first synthetic operon allowing expression of a downstream overlapping gene by translational coupling in chloroplasts. Overlapping genes provide a new mechanism for co-ordinating the translation of foreign proteins in chloroplasts.

**Electronic supplementary material:**

The online version of this article (doi:10.1186/s12870-016-0857-6) contains supplementary material, which is available to authorized users.

## Background

Methods to edit genes based on programmable nucleases have revolutionised the manipulation of nuclear genomes in multicellular eukaryotes [1, 2]. They allow precise targeted changes ranging from single nucleotide alterations to the seamless insertion of exogenous genes into nuclear chromosomes [3–5]. Successful editing gives rise to organisms with precise genome modifications, which can be free of all excess DNA such as marker genes and vector backbone sequences. Technologies to edit genomes address concerns associated with the imprecision of standard transformation technologies [6] including the contribution of excess DNA to phenotype. These advantages of genome editing have raised questions on the need to change the regulatory landscape for crops improved by transformation [7].

Outside the nucleus, important sets of genes are present in mitochondria and chloroplasts [8]. These extra-nuclear genes play essential roles in respiration, photosynthesis and development [8, 9] and are targets for improving crop productivity to ensure global food security [10]. Whilst programmable nucleases have been imported into mitochondria to induce double strand DNA breaks [11], genome editing requires the additional step of introducing a nucleic acid template into organelles to repair and introduce the desirable changes at the break sites. This requires methods that lead to the isolation of stable organelle transformants. In multicellular eukaryotes, these protocols are available for chloroplasts [12–14] but not mitochondria. Key chloroplast editing targets include the *rbcL* gene encoding the catalytic large subunit of ribulose bisphosphate carboxylase/oxygenase (Rubisco LS), the primary CO_2_ fixing enzyme, which is a focus for improvement [15–17]. Application of editing technologies to transgenes would allow their seamless insertion into chloroplast DNA to improve photosynthesis [18] and stress tolerance [19] as well as express industrial and health care products in chloroplasts [14, 20–22].

Homology dependent repair is a key component of genome editing and was used without programmable nucleases in early approaches to edit complex eukaryotic genomes [1]. The process was relatively inefficient due to low rates of homologous recombination [1]. The predominance of homologous recombination in chloroplasts [23] is a considerable advantage for developing genome editing technologies. It removes the need for making double strand breaks at editing sites with programmable nucleases [1, 2] to stimulate homologous recombination. This allows seamless insertion of selectable antibiotic and herbicide resistance genes [12, 13, 24–26] into chloroplast genomes (cp genomes). However, existing genome engineering methods to introduce point mutations or non-selectable foreign genes into the chloroplasts of wild type (WT) crops leave excess DNA associated with the transformation process, such as marker genes, or following marker excision, the target sites of site-specific recombinases and ectopic direct repeats [12–14, 24]. Seamless chloroplast genome editing methods would allow, in principle, the isolation of transplastomic derivatives that differ from the parental crop used for transformation by a single base mutation in a key chloroplast gene. Such methods need to overcome the dual challenges of the high copy number of cp genomes, which are present in thousands of copies per cell [23, 27], and reversion of edited changes by copy correction [28] with the vast excess of unedited genomes resident in WT chloroplasts. These issues have raised questions on the feasibility of isolating plants with a uniform (homoplasmic) population of edited cp genomes [4]. The possibility of using gene drive technologies [29] in organelles to address the problem of editing multi-copy genomes raises safety concerns related to containment [30].

Here we describe a two-step method to edit chloroplast genes that involves replacing the unedited WT sequence with a transient editing intermediate, which was then resolved by homologous recombination to the final edited gene. The method is applicable to angiosperm cp genomes and was exemplified in tobacco, which contains a typical 156 kb cp genome [8, 13, 31] and is the model plant for transplastomic research. The tobacco cp genome encodes about 80 polypeptides, many of which are expressed from operon-like gene clusters [8, 32, 33]. The versatility of the method was illustrated by deleting a single nucleotide from the C-terminal coding region of the *rbcL* gene in a construct containing overlapping reading frames for the *rbcL* and *gusA* [34] genes. The recombination event involved in the protocol provided a powerful tool to examine the effect of switching upstream sequences on overlapping gene expression within a single transplastomic line. Efficient translation of *gusA* demonstrated the capacity of the chloroplast translational apparatus to express overlapping foreign genes *in planta*.

## Results

### Chloroplast genome editing scheme

**Fig. 1:**
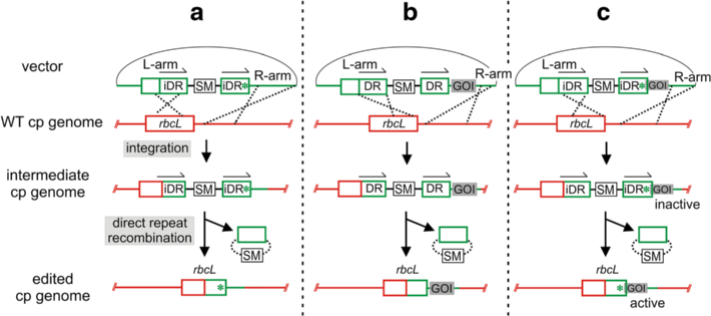
Two-step editing scheme. **a** The vector contains an imperfect direct repeat (iDR) of a partial *rbcL* sequence (*arrows*) separated by a selectable marker (SM). The right iDR contains a point mutation (*). Homologous recombination mediates integration via the left (L) and right (R) targeting arms and gives rise to the intermediate cp genome shown. For simplicity recombination between the right iDR and *rbcL* gene is not shown (see Fig. 3). Recombination between the repeated sequences excises the SM and gives rise to the edited cp genome containing a point mutation. **b** and **c** show variations that enable alternative edited products. In **b** a gene of interest (GOI) is inserted into the cp genome using a vector with perfect direct repeats (DR). In **c** a scheme for inserting a mutation and a GOI is shown. In this example the GOI overlaps a mutant *rbcL* gene. Translation coupling between the upstream *rbcL* gene and the overlapping GOI will result in GOI expression in the edited cp genome but not in the intermediate where the partial upstream *rbcL* gene forming the iDR is not translated. Not to scale

Figure 1 provides a simplified scheme to illustrate our approach to edit cp genomes using the *rbcL* gene as an example. The vector contains a 618 bp duplication of the C-terminal part of the *rbcL* gene flanking a selectable marker (SM). Insertion of a point mutation denoted by an asterisk (*) in the right *rbcL* sequence duplication creates an imperfect directly repeated (iDR) sequence (Fig. 1a). Following transformation of the vector into chloroplasts, cross-over events in the left (L) and right (R) targeting arms give rise to the intermediate cp genome containing *rbcL* iDRs. Selection for the marker results in replacement of WT cp genomes by the intermediate cp genome. This step of removing resident WT cp genomes is essential for editing multi-copy genomes. Release of selection promotes accumulation of marker-free cp genomes with the edited change (Fig. 1a) following recombination between the duplicated *rbcL* iDRs. Placing the point mutation close to the right border of the iDR ensures its retention in chloroplasts following marker excision. The approach is not limited to single point mutations and allows the seamless insertion of a gene of interest (GOI; Fig. 1b) into cp genomes using perfect direct repeats (DR). The versatility of the method is illustrated by the design used here where a point mutation in the native *rbcL* gene was combined with an overlapping *gusA* gene, chosen as the GOI. In this configuration *gusA* was only expressed following the recombination event between iDRs (Fig. 1c) because its expression was coupled [35–38] to translation of the overlapping upstream *rbcL* sequence. This allowed us to monitor the production of edited cp genomes. Any biased copy correction between the imperfect *rbcL* repeats in favour of restoration of the WT sequence would hinder the editing method.

### C-terminal *rbcL* frameshift mutation

**Fig. 2:**
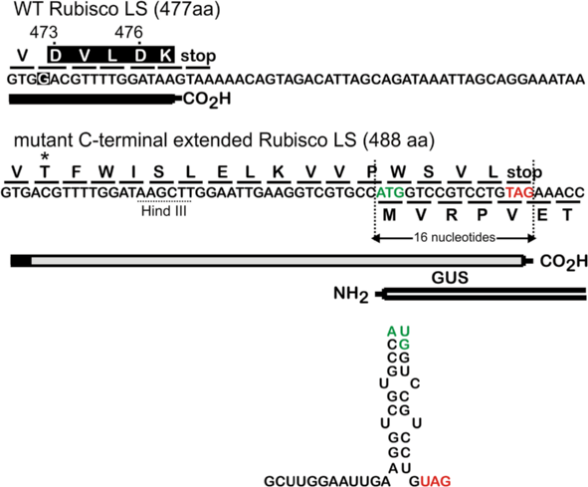
WT (*top*) and mutant (*bottom*) C-terminal Rubisco LS sequences. Deletion of a guanine in the WT *rbcL* sequence (*top*) changes aspartic acid residue 473 and all subsequent amino acids in the extended C-terminus of the encoded Rubisco LS protein (*bottom*). The ATG start codon of *gusA* and TAG stop codon of the mutant *rbcL* gene are separated by ten nucleotides. The sequence between *rbcL* and *gusA* is a synthetic linker sequence that contains a Hind III site. A predicted RNA secondary structure [41] containing the *gusA* start codon is shown

Deletion of a guanine located 15 nucleotides upstream from the TAA stop codon of the *rbcL* gene created a frameshift which altered and extended the C-terminal coding region (Fig. 2). The new reading frame ended in a TAG stop codon contained within a downstream *gusA* gene and replaced the five C-terminal residues of Rubisco LS with 16 alternative amino acids (Fig. 2). The consequences of changing the C-terminal amino acids of Rubisco LS, which is involved in homodimer interactions and formation of the active site [39, 40], had not been previously studied in higher plants. The C-terminal extended *rbcL* coding sequence, including the TAG stop codon, overlapped with the N-terminal coding region of the *gusA* gene by 16 nucleotides. In this context, the mutant Rubisco LS was encoded by reading frame one and ß-glucuronidase (GUS), the 68 kDa product of the *gusA* gene, by reading frame 3 (Fig. 2). The sequence joining the *rbcL* and *gusA* genes was a synthetic linker containing a Hind III site that is not found in the chloroplast genome. RNA fold [41] predicted sequestration of the AUG start codon of the *gusA* coding sequence in a region of secondary structure (Fig. 2).

### Step one: isolation of transplastomic plants containing the iDR intermediate cp genome

**Fig. 3:**
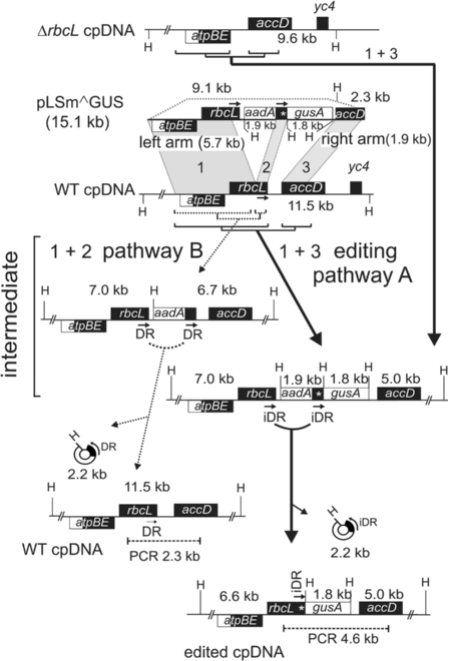
Maps showing integration of the pLSm^˄^GUS vector to form the iDR intermediate and resolution to the edited marker-free cp genome. Pathway A: desirable cross-over events in shaded regions 1 and 3 between the vector pLSm˄GUS and WT chloroplast DNA (cpDNA) integrate the iDRs which are resolved to the edited genome following iDR recombination. Integration into Δ*rbcL* cpDNA (*top*) is by pathway A only. Pathway B: undesirable cross-over events in regions 1 and 2 lead to integration of *aadA* alone and create perfect direct repeats (DR) which recombine to restore the WT cp genome. Shown are gene locations, Hind III sites (H) and fragment sizes, targeting arm sizes and locations of PCR products. Genes transcribed *left to right* are placed above genes transcribed *right to left*

The pLSm^GUS vector (Fig. 3) containing the *rbcL* iDR was transformed into the chloroplasts of WT plants by particle bombardment of leaves followed by antibiotic selection for the *aadA* selectable marker gene. The left 5.7 kb and right 1.9 kb targeting arms in the vector integrate the foreign sequences into chloroplast DNA by homologous recombination. The guanine deletion was located 14 bases from the right border of the right iDR. Two integration pathways are possible in WT chloroplasts (Fig. 3). Integration pathway A involving crossover regions 1 and 3 corresponding to the left and right arms leads to integration of *aadA*, *gusA* and the iDR. Pathway B represents undesirable integration mediated by cross-over events 1 and 2 (in the iDR), which inserts *aadA* but excludes the *gusA* gene from the cp genome. pLSm^GUS was also transformed into Δ*rbcL* mutant plants [42]. The absence of the *rbcL* gene in these plants restricts transgene integration to pathway A (Fig. 3, top), eliminating undesirable cross-over events during integration. In the wild-type background, pathway A leads to integration of an iDR intermediate in the cp genome. Removing antibiotic selection allows resolution to the final products by iDR mediated excision of *aadA* as a 2.2 kb circle. This results in the accumulation of edited cp genomes from pathway A transformants. For pathway B this results in excision of *aadA* and restoration of the WT cp genome.

**Fig. 4:**
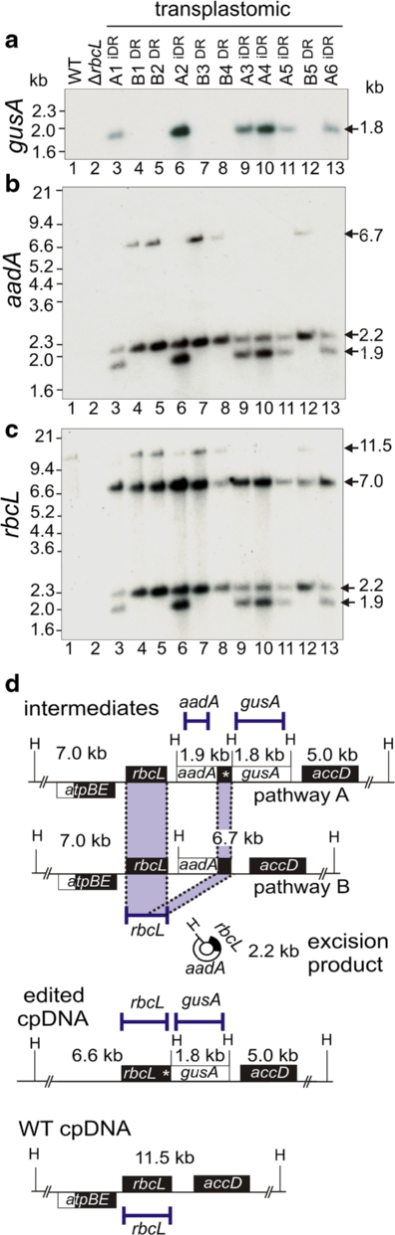
DNA blot analysis of representative intermediate transplastomic plants following pLSm˄GUS vector integration into WT plants. Transplastomic plant lines A1-6 contain imperfect direct repeats (iDRs) and lines B1-5 contain perfect direct repeats (DR). Hind III digests of total DNA from the indicated lines probed with *gusA* (**a**), *aadA* (**b**), and *rbcL* (**c**). MW standards and band sizes are indicated. Maps showing locations of probes, Hind III sites (H) and hybridizing bands (**d**)

Eleven antibiotic-resistant transplastomic plants were isolated from independent transformation events (T0 generation). T0 transplastomic plants resembled WT plants in their uniform green phenotype. DNA blot analyses on Hind III digests showed integration of transgenes by pathway A in six (A1^iDR^-A6^iDR^) and pathway B in five (B1^DR^-B5^DR^) T0 plants (Fig. 4). The hybridization patterns are shown in Fig. 4a-c and the probe binding locations in Fig. 4d. Pathway A gave rise to integrated 1.8 kb *gusA* and 1.9 kb *aadA* bands (Fig. 4a-b, lanes 3, 6, 9, 10, 11, 13). An *rbcL* probe verified the predicted 7.0 and 1.9 kb transgenic bands (Fig. 4c, lanes 3, 6, 9, 10, 11, 13). A 2.2 kb band corresponding to the linearized excised circle containing the *rbcL* direct repeat and *aadA* gene (see Figs. 3 and 4d) was detected in Hind III digests from all plants (Fig. 4b-c, lanes 3–13). This reflects spontaneous excision mediated by the directly repeated sequences. An additional three transplastomic plants from pathway A (A7^iDR^-A9^iDR^) were isolated by transforming Δ*rbcL* plants with pLSm^GUS, which was confirmed by DNA blot analysis (Additional file 1: Figure S1). Integration of transgenes by pathway B gave rise to the predicted 6.7 kb *aadA* and 7.0 kb *rbcL* bands in DNA digests from transformants B1^DR^-B5^DR^ (Fig. 4b-c, lanes 4, 5, 7, 8, 12. All pathway B plants lacked the *gusA* gene (Fig. 4a lanes 4, 5, 7, 8, 12). The 11.5 kb WT band (Fig. 4c, lane 1) was clearly visible in some pathway B lanes (Fig. 3c, lanes, 4, 5 and 7), where *aadA* excision restores the WT sequence (see Fig. 3). Incomplete replacement of WT cp genomes would also give rise to this 11.5 kb WT band and might explain the presence of this faint band in plant A2 DNA digests (Fig. 4c, lane 6). The WT lane was loaded with a lower amount of total DNA.

### Step 2: isolation of marker-free transplastomic plants homoplasmic for the edited cp genome

**Fig. 5:**
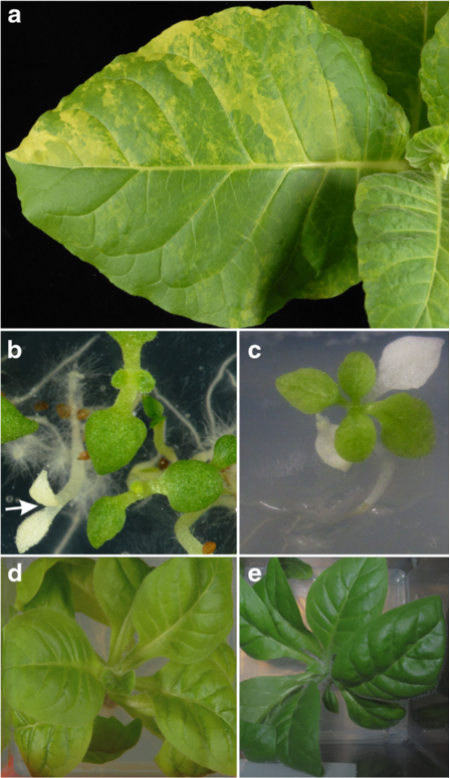
Phenotype of plants. **a** Pale-green sectors in leaves of T1 plants containing the iDR intermediate following release of selection. Sectors are linked to iDR recombination and provide a visual illustration of the process. **b** T2 Seedlings from sectoring T1 plants germinated on spectinomycin medium. White *aadA-*free seedling is arrowed. **c** A white *aadA*-free seedling transferred to antibiotic free medium produces new pale-green leaves. **d** A marker-free T2 plant containing the edited cp genome exhibits a pale-green phenotype compared to (**e**), a WT plant

Seeds were collected from the nine *gusA*-containing transplastomic lines (A1^iDR^-A9^iDR^). The T1 seed generation from all nine lines gave rise to variegated T1 plants with prominent pale-green leaf sectors when grown in soil in the absence of selection (Fig. 5a). The pale-green sectors were indicative of the phenotype associated with the mutant Rubisco LS protein (see below and Fig. 5d). The large number of pale-green sectors in all plants illustrated the high frequency of retention of the mutant *rbcL* allele following marker excision. T2 seedlings with edited genomes lacking the *aadA* marker gene were identified by their sensitivity to spectinomycin (Fig. 5b). Following recovery on antibiotic-free media (Fig. 5c) these exhibited pale-green leaves (Fig. 5d) relative to WT plants (Fig. 5e) and were propagated on sucrose medium in vitro. No sectors indicative of heteroplasmy were visible in the pale-green plants.

**Fig. 6:**
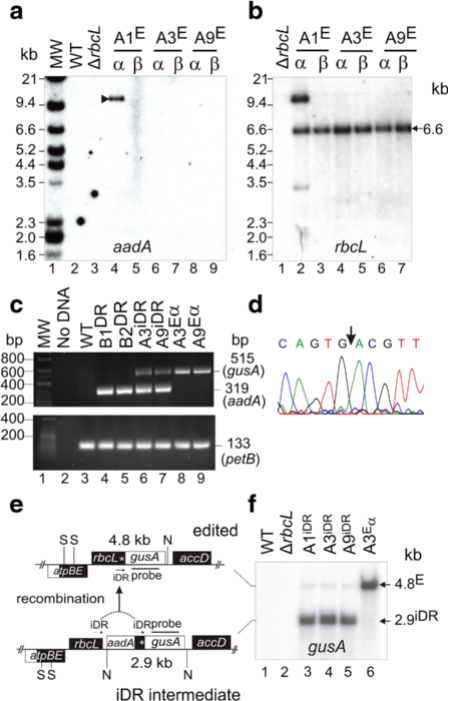
Molecular analysis of representative transplastomic plants containing the edited cp genome. Southern blot containing Hind III digests of DNA from indicated lines probed with **a** *aadA* and **b** *rbcL*. **c** PCR analyses on DNA from shown lines using primers against *aadA*, *gusA* and *petB* genes. MW standards and band sizes are indicated. **d** Sequence of A3^Eα^ *rbcL* gene showing position of deleted guanine (*arrowed*). **e** Maps of edited and iDR intermediate cp genomes showing Sac I (S) and Not I (N) sites. **f** Not I plus Sac I digests from the indicated lines probed with *gusA*. Bands from edited and iDR intermediate cp genomes are indicated

Six plants were chosen for DNA blot analysis following their random selection from the marker-free lines isolated. These were labelled α and ß for three edited transplastomic lines (A1^E^, A3^E^, A9^E^). Probes for *aadA* and *rbcL* confirmed marker excision in five of the six lines studied. Hind III restriction enzyme digests of DNA from these five lines did not bind to the *aadA* probe (Fig. 6a, lanes 5–9). They gave rise to the expected 6.6 kb *rbcL* band (Fig. 6b, lanes 3–7) containing the Hind III site shown in Fig. 2 and lacked the 1.9 kb *rbcL* iDR band and 2.2 kb Hind III linearized circle present in antibiotic resistant iDR plants (see Fig. 4c, lanes 3, 6, 9, 10, 11, 13). One line (A1^E^α) contained *aadA* and *rbcL* bands of unexpected sizes, indicating unforeseen recombination events (Fig. 6a lane 4, Fig. 6b lane 2). PCR analyses were carried out on two randomly chosen plant lines resulting from integration pathways A and B (see Fig. 3). The *aadA* gene was detected in antibiotic resistant pathway B (Fig. 6c upper panel, lanes 4–5) and pathway A (Fig. 6c upper panel, lanes 6–7) plants containing the cpDNA intermediates but not in plants containing the marker-free edited cp genome (Fig. 6c upper panel, lanes 8–9). The *gusA* gene was detected in intermediate and edited plants derived from pathway A (Fig. 6c upper panel, lanes 6–9) but not pathway B plants (Fig. 6c upper panel, lanes 4–5). WT plants did not give rise to *aadA* or *gusA* PCR products (Fig. 6c upper panel, lane 3). The chloroplast *petB* gene served as a positive control and was detected in DNA samples from all the lines tested (Fig. 6c lower panel, lanes 3–9). A 4.6 kb PCR product (location shown in Fig. 3) was purified and sequenced from three pathway-A plants: A1^E^β, A3^E^α and A9^E^α. All three plants contained the C-terminal frameshift mutation. An example is shown in Fig. 6d. The 2.3 kb PCR product from pathway-B plants lacked the mutation (not shown).

Whilst Hind III digests confirmed homoplasmy of the predicted edited cp genome in five of the marker-free T2 plants tested (Fig. 6a-b), they could not be used to provide a reliable estimate of the amount of edited marker-free cp genomes present in the T0 plants due to the similar sizes of the Hind III bands binding to the *rbcL* probe. The small fraction of edited marker-free genomes in uniformly green T0 transplastomic plants containing the iDR intermediate was determined by blot analyses using a *gusA* gene probe against DNA digested with NotI (N) and SacI (S). Maps of the iDR intermediate and edited genome are shown in Fig. 6e. DNA from a homoplasmic marker-free edited plant provided a control lane and gave rise to a single 4.8 kb *gusA* band diagnostic of the edited genome as predicted (Fig. 6f, lane 6). DNA digests from iDR plants showed a predominant 2.9 kb *gusA* band (Fig. 6f, lanes 3–5) diagnostic of the cp genome with the iDR intermediate (Fig. 6e). The 4.8 kb band corresponding to the edited cp genome was barely detectable in these lanes and represented less than 5 % of the signal intensity of the 2.9 kb band by phosphorimage analysis. This demonstrates the effectiveness of selection in suppressing the accumulation of edited cp genomes following spontaneous recombination between iDRs in the T0 plants containing the intermediate cp genome.

### Accumulation of *rbcL-gusA* transcripts

**Fig. 7:**
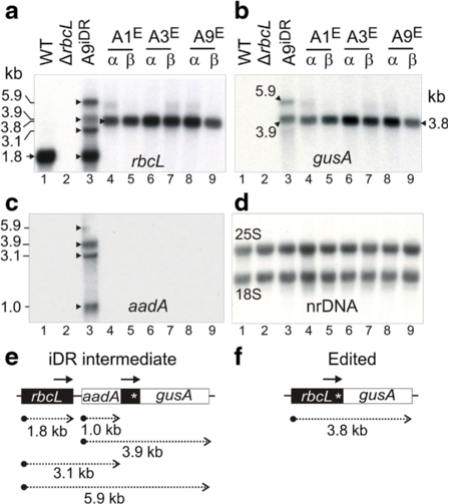
Transcript accumulation in transplastomic plants with iDR intermediate and edited cp genomes. Blots of RNA from the indicated lines probed with *rbcL* (**a**), *gusA* (**b**), *aadA* (**c**), nuclear ribosomal DNA ‘nrDNA’ (**d**). Band sizes are indicated. Maps showing locations of transcripts in plants with iDR intermediate (**e**), and edited cp genomes (**f**). *Arrows* above *rbcL* indicate iDRs

A 3.8 kb *rbcL*-*gusA* transcript present in plants with edited cp genomes hybridised to *rbcL* (Fig. 7a, lanes 4–9) and *gusA* (Fig. 7b, lanes 4–9) probes (see map in Fig. 7f). The *gusA* probe detected two transcripts of 3.9 kb and 5.9 kb in plants containing the iDR intermediate (Fig. 7b, lane 3), corresponding to transcription from the *aadA* (*rrn*) and *rbcL* promoters, respectively (Fig. 7e). The map in Fig. 7e shows the origin of the multiple transcripts containing *rbcL* (Fig. 7a, lane 3) and *aadA* (Fig. 7c, lane 3) in RNA from plants containing the iDR-intermediate. The 1.8 kb *rbcL* transcript found in WT RNA (Fig. 7a, lane 1) was not detected in edited plants (Fig. 7a, lanes 4–9) and the Δ*rbcL* negative control (Fig. 7a, lane 2). RNA loadings were normalised using a nuclear ribosomal DNA probe (Fig. 7d). Quantitative phosphorimage analysis of *gusA* hybridization indicated that the 3.9 kb transcript in iDR intermediate plants containing *aadA*, the *rbcL* repeat and *gusA* accumulated to approximately 50 % lower levels than the 3.8 kb *rbcL-gusA* mRNA present in edited plants.

### Translational coupling between *rbcL* and *gusA*

**Fig. 8:**
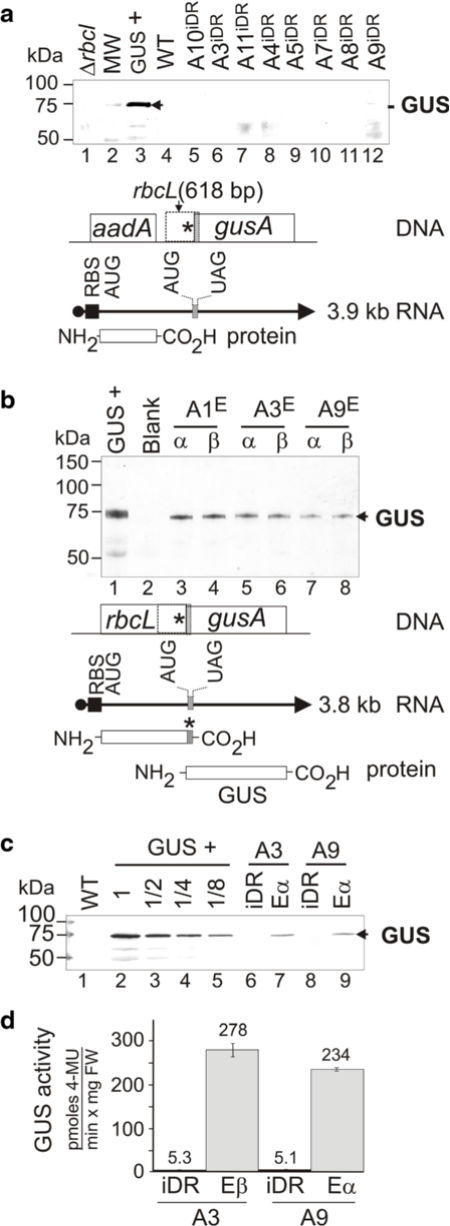
GUS accumulation in transplastomic plants with iDR intermediate and edited cp genomes. Blots of total leaf protein from the indicated lines incubated with a GUS-specific antibody (**a**-**b**). Diagrams show no translation of the upstream overlapping *rbcL* sequence, located between the *gus*A start and *rbcL* UAG termination codons, in (**a**), and translation of this sequence in (**b**). Dilutions of a positive control (GUS+) transplastomic plant accumulating GUS to 5 % of total leaf protein were used to estimate relative amounts (**c**). MW size standards and band sizes are indicated. GUS activities in leaf extracts (**d**). Average of three biological replicates showing one standard deviation. 4-methylumbelliferone (4-MU), Fresh weight (FW)

Expression of the *gusA* gene was dependent on an intact upstream gene that overlapped with it (see Fig. 2). This was comprised of the 1464 nucleotide *rbcL* gene containing the C-terminal mutation. The *gusA* gene was transcribed (see above and Fig. 7) in intermediate and edited plants and located 1.8 kb or 1.7 kb from the 5′ end of the bicistronic mRNAs, respectively. In both sets of plants, the *gusA* gene was preceded by an identical overlapping 618 bp *rbcL s*equence corresponding to the iDR. This 618 bp *rbcL* sequence was itself located downstream of a non-overlapping *aadA* gene that was translated in the intermediate iDR plants (scheme shown in Fig. 8a). An in-frame TAA stop codon was found 48 nucleotides upstream of the ATG start codon of *gusA*. No in-frame ATG or GTG codons were found in this 48 nucleotide sequence excluding alternative start sites in this region that could have led to GUS translation. In iDR plants, the *rbcL* reading frame overlapping *gusA* was not translated due to deletion of 813 nucleotides including the initiation codon from the 5′ region of the *rbcL* gene. In edited plants, restoration of the *rbcL* reading frame enables translation into the overlapping downstream *gusA* gene (scheme shown in Fig. 8b).

Protein blot analyses with a GUS-specific antibody showed a prominent 68 kDa GUS band in GUS+ transplastomic plants (Fig. 8a lane 3) expressing *gusA* under the control of chloroplast *rrn* promoter and the bacteriophage T7 gene 10 ribosome binding site [21]. A GUS band was not detected in gel-fractionated total leaf protein from intermediate iDR plants on protein blots (Fig. 8a, lanes 5–12) or the Δ*rbcL* and WT negative controls (Fig. 8a, lanes 1 and 4). In total leaf protein from edited plants, the GUS protein was detected as the major band on protein blots (Fig. 8b, lanes 3–8). GUS accumulated to 5 % of total leaf protein in the GUS+ transplastomic plants. Dilutions of leaf protein from these plants showed that GUS was present at ~0.5 % of total leaf protein in plants with edited cp genomes (Fig. 8c). Co-migration of the GUS protein band in the control (Fig. 8c, lanes 2–5) and edited lanes (Fig. 8c, lanes 7 and 9) was consistent with translation initiation at the predicted ATG start codon (Fig. 2). GUS expression mediated by ribosomal frame shifting [43] was ruled out by the lack of detection of a 120 kDa Rubisco LS-GUS fusion protein. To address low GUS expression in the iDR intermediate plants, a more sensitive β-glucuronidase fluorescence assay was performed. The assay showed a ~50-fold increase in GUS activity in plants with edited cp genomes relative to iDR intermediate plants (Fig. 8d), which equates to GUS levels of ~0.01 % of total leaf protein.

**Fig. 9:**
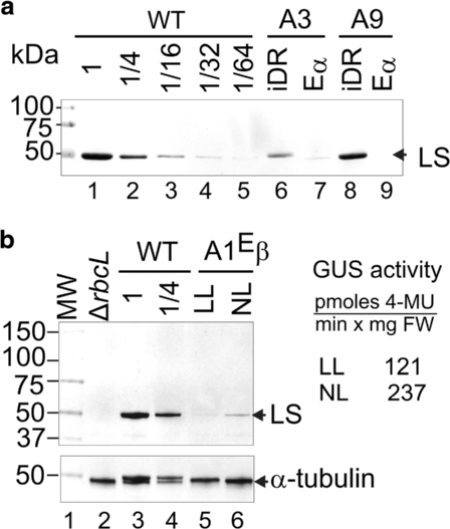
Rubisco LS accumulation in transplastomic plants with iDR intermediate and edited cp genomes. Protein blots of leaf protein from the indicated lines incubated with a Rubisco LS specific antibody. Dilutions of the WT control were used to estimate relative amounts (**a**). Blots of leaf protein from plants grown in normal light (NL) and low light (LL) incubated with Rubisco LS (*top*) and α-tubulin (*bottom*) specific antibodies (**b**). MW size standards and band sizes are indicated. GUS activities of plants grown in NL and LL are shown. Average of three biological replicates showing one standard deviation. 4-methylumbelliferone (4-MU), Fresh weight (FW)

The C-terminal modified Rubisco LS accumulated to reduced but clearly detectable levels in edited plants, which were ~30-fold lower than the amounts found in WT leaves (Fig. 9a, lanes 7 and 9). Plants with the intermediate iDR cp genome contained a WT *rbcL* gene and accumulated an abundant Rubisco LS band (Fig. 9a, lanes 6 and 8). Figure 9b shows that accumulation of the C-terminal modified Rubisco LS (lanes 5–6) and GUS activity were influenced by light intensity since they were both reduced in plants placed in low light. This is consistent with the observation that increasing light levels can positively influence Rubisco content in tobacco leaves by stimulating translation elongation on *rbcL* mRNA [44, 45]. No Rubisco LS band was detected in the Δ*rbcL* negative control lane (Fig. 9b, lane 2). The levels of α-tubulin were not markedly affected by light, lanes 2–6) and served as a loading control.

## Discussion

A two-step genome engineering scheme provided a precise and efficient method for editing the cp genome in angiosperms. The challenge of editing multi-copy organelle genomes was addressed by including a selection step to replace resident WT cp genomes with transient intermediate cp genomes. Spontaneous resolution of the iDRs in the intermediate gave rise to edited cp genomes which accumulated after release of antibiotic selection. The procedure allows the seamless insertion of point mutations and foreign genes into cp genomes. Here we deleted a single nucleotide from the *rbcL* gene which replaced the five C-terminal amino acids of Rubisco LS with 16 unrelated residues. This reduced Rubisco LS accumulation by ~30-fold relative to the WT polypeptide. The editing procedure provided a recombination switch to examine conditions required to express an overlapping *gusA* gene within the same transplastomic line. GUS accumulated to ~0.5 % of total leaf protein provided the upstream overlapping *rbcL* translation unit was complete.

The overall efficiency of our cp genome editing method was dependent on integration of the iDR intermediate into the cp genome, followed by resolution to the final edited genome triggered by internal recombination events between the iDRs. Approximately half of the transplastomic lines isolated after transformation incorporated the iDR with the point mutation. Maintenance of the iDR in transplastomic plants showed that copy correction back to the WT sequence does not take place at a frequency that prevents the isolation of edited cp genomes. The conversion of the iDR intermediate to the final edited cp genome was mediated by native chloroplast DNA recombination enzymes. The recombination event excised an unstable DNA circle containing *aadA*, which was lost, making the process unidirectional. The procedure is efficient and does not require the use of negative selection [46, 47] to promote marker excision. Following resolution of the iDR intermediate, over 80 % of the resulting marker-free plants contained the edited cp genome. Combining both steps gave an overall success rate of 40 % desirable lines isolated per total number of transplastomic lines screened. This compares favourably with nuclear genome editing where efficiencies above 0.5 % were considered successful in human cell lines [2]. The use of chloroplast deletion mutants that lack the editing target, in this case the *rbcL* gene [42, 48], increase the efficiency by two-fold by eliminating undesirable integration events.

The C-terminal mutation studied here reduced Rubisco LS accumulation by ~30-fold and may have affected biogenesis of the holoenzyme, which follows a complex pathway involving chaperones [49]. By comparison, addition of a C-terminal His-tag had little impact on Rubisco accumulation [50]. Reduction in Rubisco accumulation was associated with a light green phenotype, which allowed the recombination process to be monitored by the appearance of pale sectors in leaves (Fig. 5a). Whilst we used the *rbcL* gene, which is a focus for improvement [15, 16], to illustrate the method, the procedure is applicable to a wide selection of chloroplast genes. Our results show that recombination based editing enables deleterious mutations that reduce plant fitness to be introduced into chloroplast genes. This suggests it should be possible to introduce a wide variety of point mutations into chloroplast genes by our genome editing method. Loss of function mutations would be restricted to non-essential chloroplast genes, whose dysfunction can be rescued by growing plants on sucrose medium [14, 51]. Homologous recombination is the predominant pathway of gene integration in all species in which chloroplast transformation has been reported [12, 14]. This indicates that the two-step editing procedure described here will be applicable to cp genomes in a broad range of plant species. Moreover, because the method works on WT cells it provides a route for editing the cp genomes of obligate phototrophic algae amenable to chloroplast transformation [52].

The dependence of *gusA* expression on a complete upstream *rbcL* gene was consistent with translational coupling between the two genes. Translational coupling has been described in bacteria [35, 36, 53], bacteriophages [54], animal viruses [36, 53, 55] and appears to operate in chloroplasts, although it has only been demonstrated in vitro [37, 38, 56]. Additional file 2: Figure S2 shows the four overlapping gene pairs found in the tobacco cp genome: *atpB-atpE*, *ndhC-ndhK*, *psbD-psbC* and *rpl22-rps3* gene [31]. The results from experiments using chloroplast lysates support translational coupling at *psbD-psbC* [37], *ndhC-ndhK* [38, 56] but not *atpB-atpE* [57], which was consistent with ribosome profiling [58] but conflicts with an earlier study [59]; *rpl22-rps3* remains untested. Analysis of bacterial genes found limited conservation of the sequences required for translational coupling [60]. The overlap tested here is a synthetic sequence not found in chloroplasts. Only weak similarities between the overlapping sequences present in *rbcL-gusA* (Fig. 2) and the four pairs of native chloroplast genes (Additional file 2: Figure S2) were found. Shared features between *rbcL-gusA* and *ndhC-ndhK* include: termination of the upstream coding sequence in reading frame one by a TAG codon, and the presence of a GTC valine codon following the ATG start codon of the downstream ORF in reading frame 3. However, the *rbcL-gusA* overlap is six nucleotides longer than the ten nucleotide overlap between *ndhC* and *ndhK* (Additional file 2: Figure S2) but is the same length as the overlap found in *rpl22*-*rps3*.

In bacteria, a number of mechanisms influence translational coupling including the rate at which terminating ribosomes re-initiate translation at the upstream AUG and the influence of mRNA structure on translation [35, 36, 60, 61]. A change in RNA structure could explain the results observed here. In the uncoupled state the *gusA* start codon is sequestered by local base pairing in a folded structure (Fig. 2). Following *rbcL* translation, this folded RNA structure would be unwound allowing ribosomes to bind the region containing the *gusA* start codon and initiate translation. Absence of secondary structure appears to be a sufficient condition for recognition of start codons and initiation of translation in prokaryotic systems [62]. Other explanations could involve initiation of *gusA* translation at alternative start sites. However, no in-frame AUG start codons were found in the region upstream of *gusA*, which was preceded by a UAA stop codon located 48 nucleotides upstream of the *gusA* initiation codon. The first in-frame AUG codon within the *gusA* coding region would give rise to a shorter polypeptide of 56 kDa, which was not detected. The possibility that *gusA* was translated at similar rates in marker-free edited T2 plants and intermediate T0 plants but that the resulting GUS protein was only stable in T2 plants appears unlikely given the many reports of stable GUS expression in chloroplasts [63, 64]. Further insights into the detailed mechanism of translational coupling will require an analysis of *gusA* translational activity, through methods such as ribosome profiling [58], radiolabelling of translation products [65] and analysis of mRNA bound to polysomes [66].

To-date multiple proteins have been expressed in chloroplasts from constructs containing non-overlapping genes [67, 68]. Translational coupling provides a tightly controlled mechanism to regulate the stoichiometry of proteins expressed from overlapping genes [55]. Successful expression of an overlapping *gusA* transgene in chloroplasts provides a new approach for expressing multiple proteins from polycistronic transcripts in chloroplasts and is applicable to expressing multi-subunit complexes, such as carboxysomes [17, 69] and novel metabolic pathways [68], in chloroplasts.

## Conclusions

An important set of genes is located in chloroplasts including those essential for photosynthesis such as *rbcL*. These genes are key targets for improving crop productivity to address global food security in the era of climate change but are beyond the reach of current nuclear genome editing technologies. Here we have described an efficient and versatile method to edit angiosperm chloroplast genomes. It is based on the native homologous recombination pathway acting on transient imperfect direct DNA repeats. The method unlocks the potential of organelle transformation to produce a new class of biotech crops, which differ from their parental cultivars by single base mutations in the chloroplast. The method was used to show that the C-terminal residues of Rubisco LS are important for its accumulation. Using the bacterial *gusA* gene we have shown, for the first time that a foreign protein can be expressed from overlapping chloroplast genes *in planta*. Sequestration of the initiation codon of the downstream *gusA* gene in secondary structure predicted by RNA fold [41] provided a mechanism for explaining the observed translational coupling between *gusA* and the upstream *rbcL* gene. The overlapping sequence used was entirely synthetic indicating that translational coupling is not restricted to native sequences found in chloroplasts. Translational coupling and overlapping genes provide a new approach for co-ordinating the expression of foreign proteins in chloroplasts.

## Methods

### Recombinant DNA procedures

Standard procedures for manipulation of plasmids and transformation of *Escherichia coli* Solo Pack Gold (Agilent technologies, Stockport, UK) were used to construct pLSm^GUS. The 618 bp *rbcL* DR sequence was amplified as a 0.64 kb product with primers 410-F (5′GGGGGGATTCACCGCAAATA) and 418-R (5′CCATGGCACGACCTTCAATTCCAAGCTTATCCAAAACGTCCACTGCT) using pATB27-link template [21] and inserted into pGEM-T easy (Promega, Madison, USA). A deletion of a C (under-lined) in the 418-R primer binding region in a PCR product cloned into pGEM-T created the frameshift mutation. The 0.66 kb insert with the *rbcL* iDR was released with NotI and NcoI from pGEM-T easy and ligated to the 5′ end of a plasmid containing *gusA* linked to the 3′ UTR of *Chlamydomonas reinhardtii rbcL*. The resulting 2.9 kb *rbcL* iDR repeat-*gusA*-3′UTR sequence was excised with NotI and used to replace the TGFβ3 expression cassette in p201 [21] giving rise to pLSm˄GUS. Following chloroplast transformation, the 4.2 kb foreign sequence containing *aadA* and *gusA* genes was integrated after base 59,328 of the 155,943 nucleotide *N. tabacum* cp genome (Accession Z00044.2).

### Isolation and propagation of chloroplast transformants

*Nicotiana tabacum* cv. Wisconsin 38 was used for all experiments. WT seeds were obtained from Mr. Thurston Heaton (Firs Experimental Gardens, Manchester, UK) and the Δ*rbcL* mutant derived from WT plants as described in Kode et al., 1986 [42]. Chloroplast transformation experiments on WT plants were carried out as previously described [24] using three cycles of regeneration of resistant shoots on medium containing spectinomycin (500 mg/L) and streptomycin (500 mg/L). Only one cycle of regeneration on both antibiotics was required when using leaves from the *Nicotiana tabacum* cv. Wisconsin 38 Δ*rbcL* mutant [42]. Plants were grown in a 12 h day at 25 °C with light intensities of 130–200 μE m^−2^ s^−1^ at 25 °C. For low light conditions, plants were grown in 30 μE m^−2^ s^−1^ light at 25 °C. Marker-free seedlings identified by bleaching on 100 mg/L spectinomycin MS medium were transferred promptly to antibiotic free MS medium to recover.

### PCR amplification and sequencing

Total leaf DNA was used as template with primers EXTrbcLOutDR-F (5′-CCGAAGCACTTTATAAAGCACAGGCTGA) and EXTrbcLOutDR-R (5′- AATCCATAACCCCAATTGCTACGG), to amplify a 4.6 kb PCR product from edited lines and a 2.3 kb PCR product from DR intermediate lines (see Fig. 3). Gel purified PCR products were sequenced using primer INTrbcLmut-F (5′-TGTAAAAGCTCGTAATGAAGGACGTGA the BigDye® Terminator 3.1 Cycle Sequencing Kit (Applied Biosystems) and fractionating ladders on a 3730 Genetic Analyser (Applied Biosystems) in the DNA Sequencing Facility at The University of Manchester. Primers [24] CGTCATCGAGCGCCATCTCGAA (aadA-F) and TGGCTCGAAGATACCTGCAAGAAT (aadA-R), GCGTTACAAGAAAGCCGGGCAAT (gusA-F) and TTGGCCACCACCTGCCAGTCAA (gusA-R), GTATCTCACCGGCGGATTTA (petB-F) and CACTGCCCAATAACCGACTTT (petB-R) were used to detect *aadA*, *gusA* and *petB* genes in total DNA from plants by PCR using Biomix Red (Bioline, London) with conditions of 95 °C for 60 s followed by 26 cycles of: 94 °C, 30 s; 58 °C, 30 s, 72 °C 45 s.

### Nucleic acid analyses

Total leaf DNA and RNA extractions and blot analyses were carried out as previously described [70] and digested with restriction enzymes according to the supplier (New England Biolabs). Hybridization probes were gel purified PCR products using the following plasmid templates and primers: 1.4 kb *rbcL* probe, TOBrbcL*-*F (5′- ATGTCACCACAAACAGAGACTA) and TOBrbcL-R (5′- TTACTTATCCAAAACGTCCACT) with pATB27-link [21]; a 0.6 kb *aadA* probe, ELLE-aadA-F (5′- GAAGCGGTTATCGCCGAAGTATCGAC) and ELLE-aadA-R (5′-GATTTTGCCGGTTACTGCGCTGTA) with pUC atpX *aadA* [71]; a 1.8 kb *gusA* probe, GUSAprobeF (5′-TCCGTCCTGTAGAAACCCCAACCC) and GUSAprobeR (5′-TTCATTGTTTGCCTCCCTGCTGCG) with pJD330 [72]. Following hybridization with [α^32^P]-dCTP labelled DNA probes, band intensities were quantified by phosphorimage analysis (FLA-3000 Image Analyzer, Fuji). RNA fold was used to predict secondary structures using the default parameters [41].

### Protein analyses

Total leaf protein was fractioned by SDS-PAGE, transferred to Hybond-ECL nitrocellulose membranes and incubated with monoclonal anti-Rubisco LS [73], anti-α tubulin [74] and polyclonal anti-GUS (rabbit IgG, from Molecular Probes) antibodies as described [21, 70]. Bands were visualised by using secondary antibodies conjugated to alkaline phosphatase and staining with 5-bromo-4-chloro-3-indolyl phosphate/nitro blue tetrazolium (BCIP/NBT) (Sigma-Aldrich, Poole, UK). GUS enzyme activities [34] were quantified with 4-methylumbelliferyl-β-D-glucuronide (Sigma-Aldrich) using a Synergy HT Multi-Mode Microplate Reader (BioTek Instruments). 5-bromo-4-chloro-3-indolyl β-D-glucuronide was used for histochemical staining of GUS activities in leaves.

## Abbreviations

cp genome*,* chloroplast genome; DR*,* direct repeat; GUS*,* β-Glucuronidase; iDR*,* imperfect direct repeat; LS*,* large subunit; Rubisco*,* ribulose bisphosphate carboxylase/oxygenase; SM*,* selectable marker; Δ*rbcL, rbcL* deletion mutant

## Additional files

Additional file 1: Figure S1. Analysis of iDR intermediate transplastomic plants following pLSm˄GUS vector integration into Δ*rbcL* plants [42]. DNA blot analyses of Hind III digests of total DNA from the indicated lines probed with (A) *aadA*, (B) *accD*, and (C) *gusA*. Band sizes are indicated. For map see Fig. 4d. (TIF 6312 kb) Additional file 2: Figure S2. The *Nicotiana tabacum* cp genome contains four pairs of overlapping genes: *ndhC-ndhK*, *psbD-psbC*, *atpB-atpE* and *rpl22-rps3* [31]. The overlapping regions of these genes are shown together with the N- and C-termini of the encoded proteins. (TIF 5830 kb)
