# Supplementary figures and images for: Seamless editing of the chloroplast genome in plants
Source: BMC Plant Biol. 2016 Jul 29;16:168. doi: 10.1186/s12870-016-0857-6 (PMC4966725; doi:10.1186/s12870-016-0857-6)

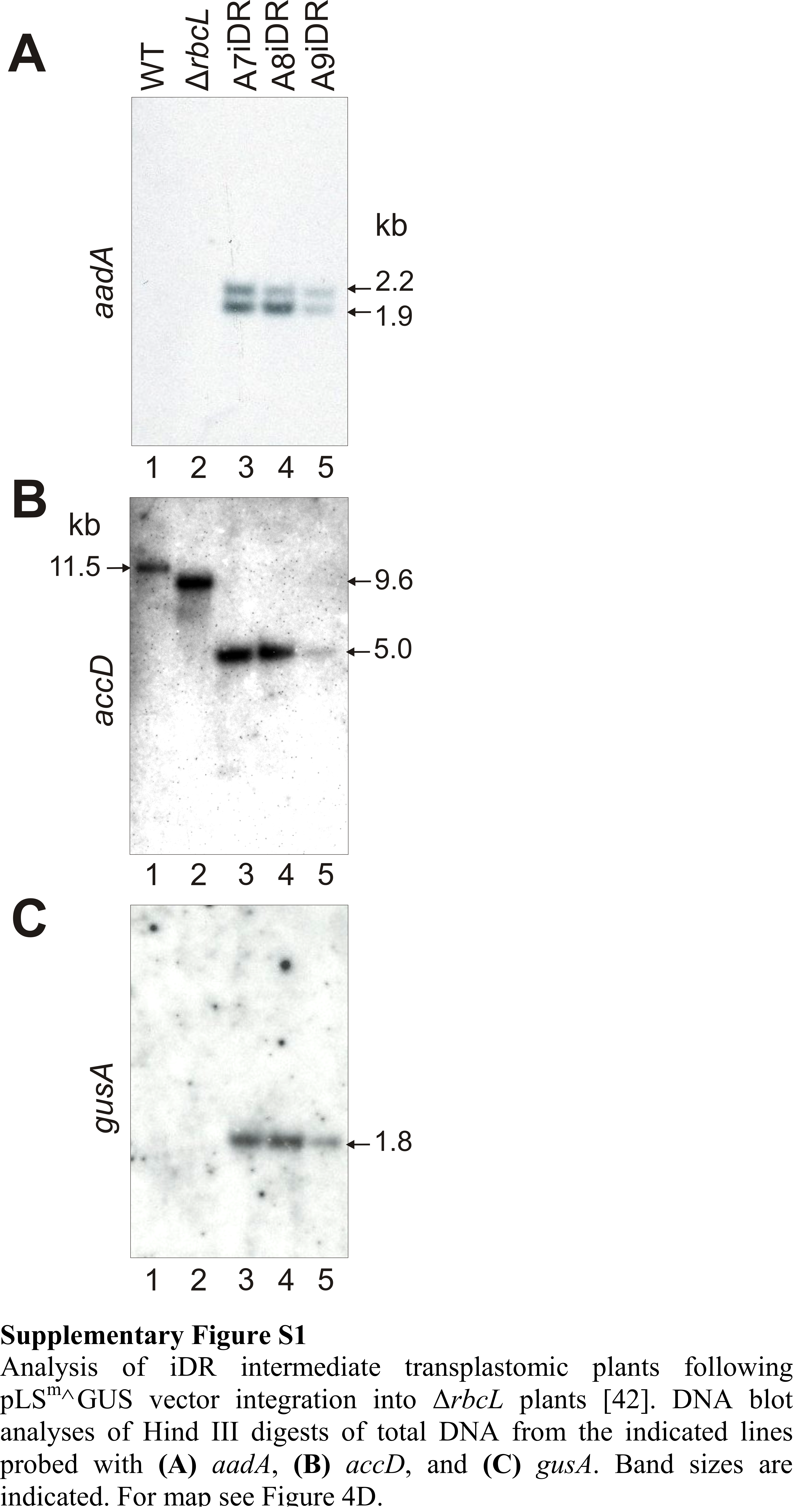

Supplement: Additional file 1: Figure S1. — Analysis of iDR intermediate transplastomic plants following pLSm˄GUS vector integration into ΔrbcL plants [42]. DNA blot analyses of Hind III digests of total DNA from the indicated lines probed with (A) aadA, (B) accD, and (C) gusA. Band sizes are indicated. For map see Fig. 4d. (TIF 6312 kb) [file 12870_2016_857_MOESM1_ESM.tif]
